# Supplementary material for: The Rift Valley fever (RVF) vaccine candidate 40Fp8 shows an extreme attenuation in IFNARKO mice following intranasal inoculation
Source: PLoS Negl Trop Dis. 2024 Aug 19;18(8):e0012011. doi: 10.1371/journal.pntd.0012011 (PMC11361746; doi:10.1371/journal.pntd.0012011)
Supplement: S1 Table — Groups of n = 8 A129 mice 8–12 weeks old were inoculated by the IN route with a low (L) or high (H) dose (50 or 1000 pfu, respectively) of the indicated viruses: 40Fp8 (S1A Table), rG1 (S1B Table) and rMP-12 (S1C Table). Each group included 4 Female (F) and 4 Male (M). Whole blood samples collected at day 3 pi were processed to test for viral load by RT-qPCR. Samples rendering a Cq value over o close to 33.0 were further assayed for infectious virus isolation (VI) by inoculation of 5–20 microliters (depending on the availability) on cell culture. A few samples were included as internal controls of the assay (M_L_3; F_L_4). Labelling of the animals is the same as in Figs 1C and 2A. Day of death for each animal is indicated. FD: found dead (in some cases blood sample could not be collected); EU: euthanized; S:survivor at day 15 pi (end of the experiment). For RT-qPCR results, the Cq given by the sample is indicated. The results of virus isolation are expressed as follows: NEG (negative, no cpe after 3 passages); (++) (very positive, cpe registered after 1st passage). The specificity of the cpe was confirmed by IFI. ND = not done. (DOCX) [file pntd.0012011.s001.docx]

**SUPPLEMENTARY TABLE 1.**

**Table 1a. Animals inoculated with RVFV 40Fp8**

| Mice ID | Day of  death | Cq | VI |
| --- | --- | --- | --- |
| MH1 | S | 32.95 | NEG |
| MH2 | S (15 EU) | 33.01 | NEG |
| MH3 | S (15 EU) | 36.97 | NEG |
| MH4 | S | 32.87 | NEG |
| FH1 | S (15 EU) | 34.00 | NEG |
| FH2 | S | 39.14 | NEG |
| FH3 | S (15 EU) | 34.39 | NEG |
| FH4 | 9 EU | 35.86 | NEG |
| ML1 | S | 38.83 | NEG |
| ML2 | S | 32.49 | NEG |
| ML3 | S | 34.46 | NEG |
| ML4 | S | 34.74 | NEG |
| FL1 | S | 36.51 | NEG |
| FL2 | S | 36.44 | NEG |
| FL3 | S | 37.85 | NEG |
| FL4 | S | 36.05 | NEG |

**Table 1b. Animals inoculated with RVFV rG1**

| Mice ID | Day of  death | Cq | VI |
| --- | --- | --- | --- |
| MH1 | 3 EU | 25.58 | ND |
| MH2 | 3 EU | 23.44 | ND |
| MH3 | 3 EU | 26.93 | ND |
| MH4 | 3 FD | ND | ND |
| FH1 | 3 FD | ND | ND |
| FH2 | 3 EU | 24.92 | ND |
| FH3 | 3 FD | ND | ND |
| FH4 | 3 EU | 26.79 | ND |
| ML1 | 4 FD | 31.56 | ++ |
| ML2 | 4 FD | 33.02 | ++ |
| ML3 | 3 EU | 22.2 | ++ |
| ML4 | 5 EU | 32.42 | ++ |
| FL1 | 4 EU | 24.12 | ND |
| FL2 | 4 FD | 21.65 | ND |
| FL3 | 4 FD | 30.29 | ND |
| FL4 | 3 FD | 21.26 | ND |

**Table 1C. Animals inoculated with RVFV rMP-12**

| Mice ID | Day of  death | Cq | VI |
| --- | --- | --- | --- |
| MH1 | 4 FD | 29.67 | ++ |
| MH2 | 4 EU | 35.66 | ++ |
| MH3 | 3 EU | 25.15 | ND |
| MH4 | 4 FD | 24.28 | ND |
| FH1 | 3 EU | 22.35 | ND |
| FH2 | 3 EU | 24.12 | ND |
| FH3 | 3 EU | 21.26 | ND |
| FH4 | 3 EU | 34.68 | ++ |
| ML1 | 5 FD | 38.18 | NEG |
| ML2 | 5 FD | 35.51 | ++ |
| ML3 | 5 FD | 40 | ++ |
| ML4 | 5 FD | 38.93 | ++ |
| FL1 | 4 EU | 28.7 | ND |
| FL2 | 4 EU | 31.46 | ++ |
| FL3 | 4 EU | 36.33 | ++ |
| FL4 | 4 FD | 27.21 | ++ |
