# Supplementary figures and images for: The Rift Valley fever (RVF) vaccine candidate 40Fp8 shows an extreme attenuation in IFNARKO mice following intranasal inoculation
Source: PLoS Negl Trop Dis. 2024 Aug 19;18(8):e0012011. doi: 10.1371/journal.pntd.0012011 (PMC11361746; doi:10.1371/journal.pntd.0012011)

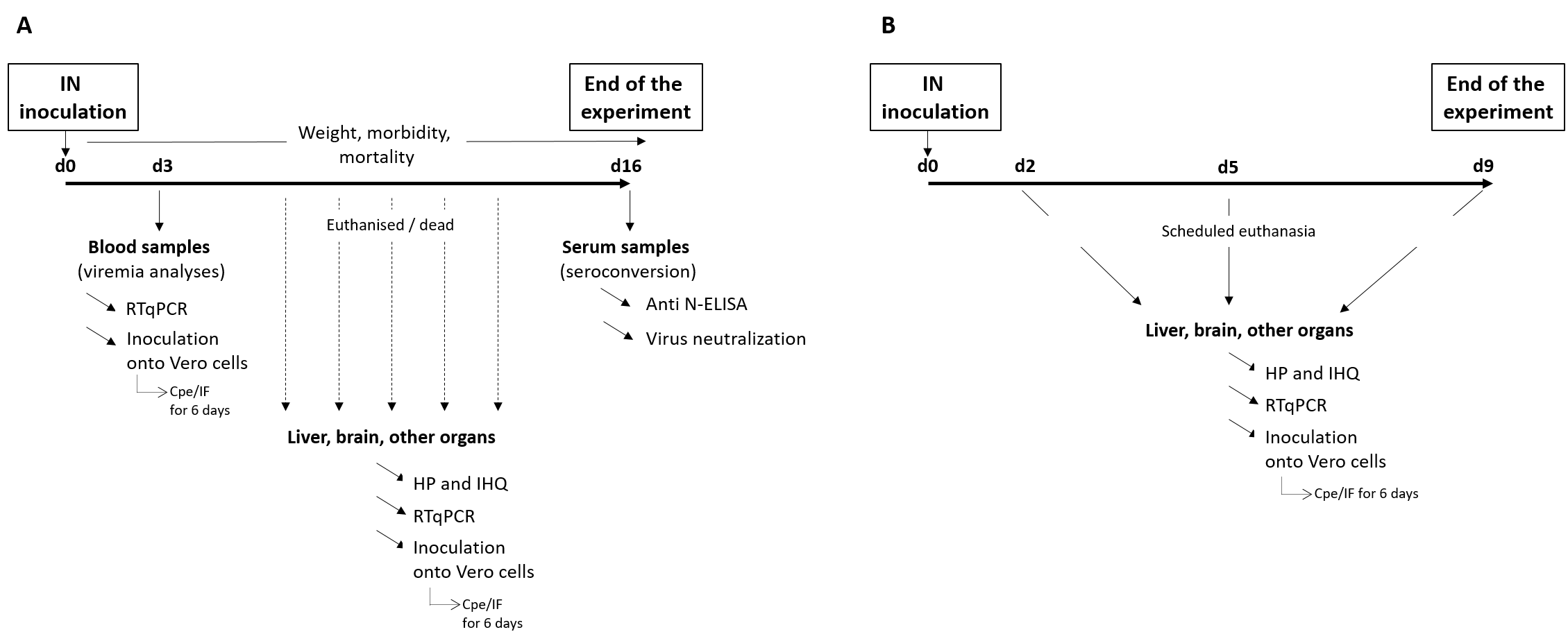

Supplement: S1 Fig — (a) Experiment 1, virulence assessment; (b) Experiment 2, pathological findings. (TIF) [file pntd.0012011.s002.tif]

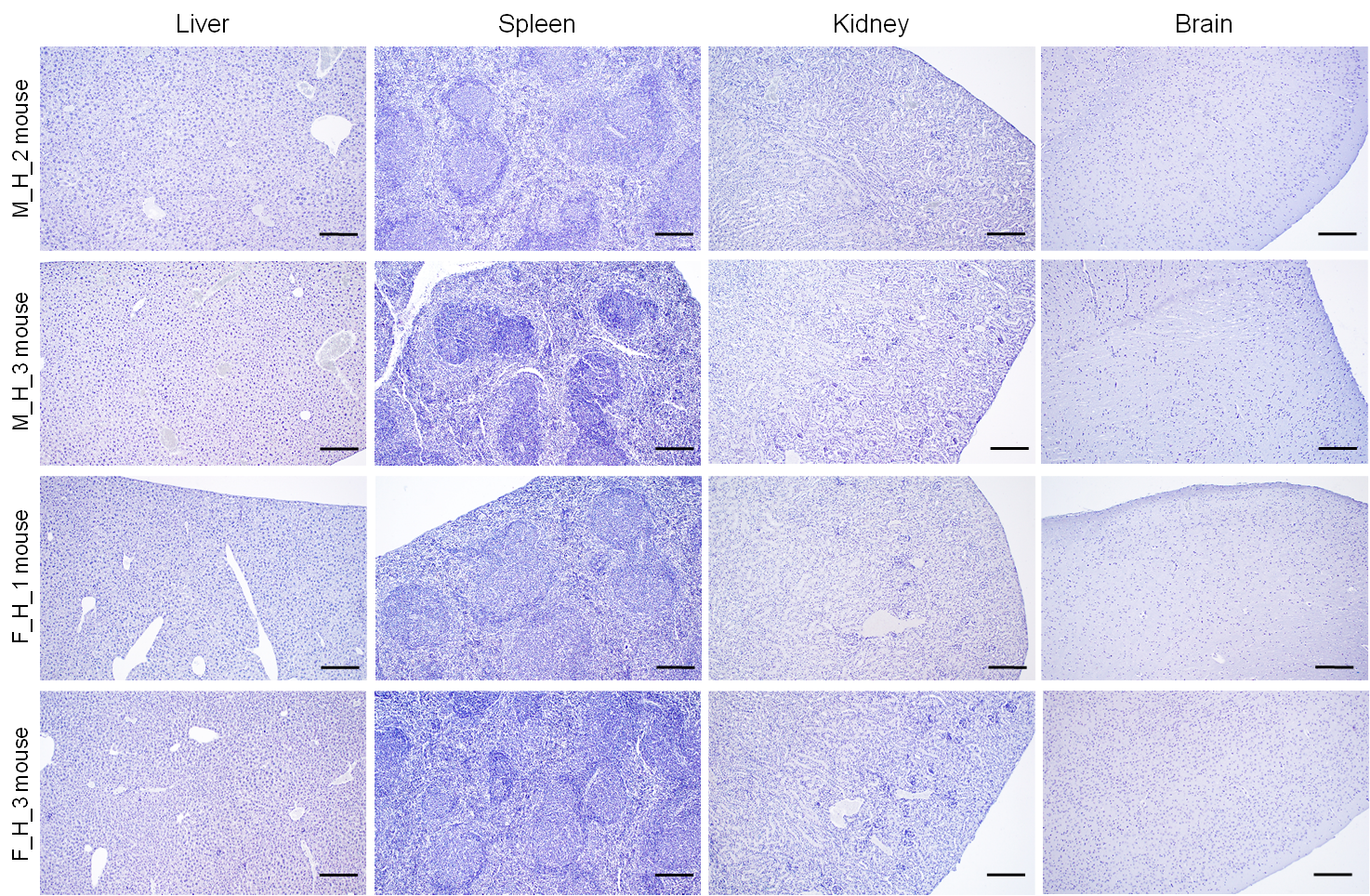

Supplement: S2 Fig — 40Fp8-inoculated mice euthanized on day 15 pi, identified by sex (M/F) and viral dose received (H/L) as in the legend of Fig 1, were selected for histopathological (HP) and immunohistochemical (IHC) studies (mice M_H_2, M_H_3, F_H_1 and F_H_3). Data for each animal are shown in S1A Table. Representative images of tissue sections (liver, spleen, kidney and brain) immunolabelled against RVF virus antigen are shown. Immunolabeled cells were not observed in any of the selected tissues. IHC, black scale bars: 200 micrometers. (TIF) [file pntd.0012011.s003.tif]
