# Supplementary material for: The Rift Valley fever (RVF) vaccine candidate 40Fp8 shows an extreme attenuation in IFNARKO mice following intranasal inoculation
Source: PLoS Negl Trop Dis. 2024 Aug 19;18(8):e0012011. doi: 10.1371/journal.pntd.0012011 (PMC11361746; doi:10.1371/journal.pntd.0012011)
Supplement: Attachment — Submitted filename: Borrego et al RESPONSES TO REVIEWERS.pdf [file pntd.0012011.s005.pdf]

Dear Editors,

We are pleased to submit our answers to the Reviewer's Questions about our manuscript entitled "The Rift Valley fever (RVF) vaccine candidate 40Fp8 shows an extreme attenuation in IFNARKO mice following intranasal inoculation", PLOS Neglected Tropical Diseases submission (PNTD-D-24-00268).

We would like to thank the reviewers for their constructive feedback on our work and results. In particular, we appreciate the insightful questions that have helped us to improve our manuscript. We hope that the revised version of our manuscript can now be considered for publication in your journal.

Yours faithfully,

Alejandro Brun and Belén Borrego

CORREO borrego@inia.csic.es

Ctra. de Algete a El Casar, s/n
28130 Valdeolmos
MADRID
TEL: 91 620 23 00
FAX: 91 620 22 47

ANSWER TO Reviewer's Questions on the manuscript "The Rift Valley fever (RVF) vaccine candidate 40Fp8 shows an extreme attenuation in IFNARKO mice following intranasal inoculation" (PLOS Neglected Tropical Diseases submission PNTD-D-24-00268)

We would like to thank the reviewers for their constructive feedback on our work and results. In particular, we appreciate the insightful questions that have helped us to improve our manuscript and address future research. We hope to have answered to them with the responses provided below.

Methods

Reviewer #1: This is a clearly written manuscript on the the Rift Valley fever vaccine candidate 40Fp8 that discusses attenuation in mice.

1. The objectives of the study are clearly articulated with a testable hypothesis
2. The study design is appropriate and the mice population for testing is clearly described
3. The statistical analyses are limited, beyond stating what software was used and the significance level for p-values, the aim and scope of the statistical tests are lacking

We thank the reviewer for the positive comments. Regarding statistical analysis, we agree these were only succinctly detailed in the figure legends for the individual experiments. In order to inform on the statistical approach followed, this is now reported in the Materials and Methods section that has been rewritten as follows:

Due to the exploratory nature of this research work, a formal hypothesis on the expected outcomes was not pre-specified. To control variation, inbred animals were used, which allows to reduce the number of animals per experimental unit using a sample size of n=8. This sample size has a 95% power to detect an effect size of 12.5 % in means, assuming equal variances, and an effect size of 0.7 in frequencies, for a 2% significance level and a two-sided test [39]. Mice were randomly distributed with 50% males and females per group. Investigators were not blinded to group's outcome assessment. All statistical evaluations were performed using the GraphPad Prism version 8.0 (GraphPad Software, San Diego, CA). Survival curves were compared using the log rank (Mantel-Cox) test. Comparison of viral RNA qRT-PCR and immune response data between groups was performed using multiple t-tests (Holm-Sidak method and Mann-Whitney test respectively). Differences were considered statistically significant when p < 0.05.

A new reference (numbered as 39 in the corrected version) has been included
Charan J, Kantharia ND. How to calculate sample size in animal studies? J Pharmacol Pharmacother. 2013;4(4):303-6. Epub 2013/11/20. doi: 10.4103/0976-500X.119726. PubMed PMID: 24250214; PubMed Central PMCID: PMCPMC3826013.

In addition, the results shown in figure 3B, which were only considered qualitatively, have now been subjected to statistical analysis and the results have been added in the figure and in the legend, where the following sentence has been added:

Statistical significance determined by Mann-Whitney test. P value: 0.0023.

Finally, some figure legends have been modified in order to provide a more accurate explanation of the results. The changes introduced are as follows:

Figure 1 (a) Survival curves. *Percentages of survival are represented using Kaplan-Meier plots. Survival distributions were compared by Log-rank (Mantel-Cox) test. The survival curves are significantly different with p <0.0001.*

In figure 3a and 3b the sentence *"bars indicate the mean value"* has been added.

4. I would have liked to see a schematic or table explaining what was observed or tested for on which days. It was difficult to work out what was checked for on each day relying only on written information in text format. For example, what was checked on each specific day: "Day 3: blood samples were taken for analysis of viremia by RT qPCR... Days 6-7: Cultures were kept in the incubator at 37º C and checked up to 6-7 days for cytopathic effect (cpe) fluorescence in the case of virus rG1), etc."

In order to clarify the experimental design regarding the inoculation of mice, and sample collection and analysis of the two trials described in the paper a new figure has been included where all the procedures are indicated (supplementary figure 1 and its corresponding legend) .

5. citation format is inconsistent throughout, i.e. why is there a website link on lines 66-67?

This was a typing mistake and has now been deleted.

Reviewer #2: Objectives clearly articulated and hypothesis clearly stated.
The study design in general was appropriate for the hypothesis tested.
The power is sufficient and statistical analysis is correct to support conclusions.

Reviewer #3: The methods are well described for all aspects of the study. I would recommend that the ethics statement (Line 171 is placed at the start of the section on "Mice inoculation and sampling" rather than the end.

Changed as suggested

Minor editing needed for reference citation, line 143.

Corrected

The statistical analysis is sufficient for the study.

Results

Reviewer #1: 1. the figures are clearly presented and captions make sense, I appreciated the explicit text re shaded regions and interpretation of the box and whiskers 2. the figures are of sufficient quality 3. I would have expected more statistical analyses beyond just stating one p-value, this would make the findings of the paper stronger

As stated above in the corrected version we have added more detail in the descriptive statistics and testing for differences between groups.

Reviewer #2: The results are clear and well presented with quality and clarity.

Reviewer #3: The analysis of the data is acceptable

Conclusions

Reviewer #1: 1. The findings of the animal models are clearly summarised, but again would have expected more on the statistical conclusions 2. There is little or no commentary on the public health relevance of this vaccine, if the vaccine were to demonstrate efficacy in animal models, what would be the next steps? Could this be a vaccine that becomes an intervention in the human population? What would be the limitations or evidence gaps for this vaccine in the human population? What can we learn from other vaccines under development or recently licensed (i.e CHIKV)? What are the considerations for next steps in the vaccine development pathway for RVF?

As discussed above, the new version of the manuscript includes more detail in the descriptive statistics and testing for differences between groups. To address the issues raised in point 2, the following paragraph has now been added at the end of the discussion section.

Rift Valley fever has been identified by the WHO R&D Blueprint as one of the top ten priority pathogens that "pose a public health risk because of their epidemic potential and for which there are no, or insufficient, countermeasures". In addition to the human impact, the repercussions in endemic areas in underdeveloped tropical countries, where livestock is the main source of income and food, are dramatic, causing important monetary losses to producers in both the short and long term. The availability of a vaccine that provides safe immunisation against RVFV, with potential use in both humans and livestock will help to address the problems derived from RVFV infection. Once demonstrated its high attenuation in an immunodeficient model, further work is underway to assess the suitability of 40Fp8 as an RVF vaccine. Studies to determine the stability and yield of 40Fp8 in cell culture, its transmissibility within vaccinated animals as well as its safety and efficacy in susceptible natural hosts such as pregnant sheep are currently in progress.

Reviewer #2: Conclusions support the data. The limitations can be discussed better. The authors do a good job in addressing the public health relevance.

Reviewer #3: The conclusions of the study are supported by the results presented, namely that the attenuated strain of RVFV 40Fp8 is highly attenuated relative to others an minimally pathogenic to interferon receptor ko mice.
The one area of concern is the unexplained death on one animal inoculated with high dose RVFV strain 40Fp8. The authors attribute this to liver dysfunction in the early stages of infection (line 451) leading to "toxic substances in the blood causing neurological signs. This is not very convincing and not supported by the data presented showing minimal virus presence in the liver compared to infection with other RVFV attenuated strains. This needs revising and a more plausible explanation for the death of this animal.

We appreciate the reviewer's comment on this point of discussion and agree that attributing the death of this mouse to liver dysfunction is not supported by the data presented. Although virulent strains of RVFV may induce liver damage leading to hepatic encephalopathy (brain dysfunction caused when the liver does not filter toxins as it should), in this mouse virus was not detected in the brain or other organ samples on day 9 pi, no signs of moderate or severe liver damage were found (Figure 2 h), and no blood chemistry was analysed. Therefore, the statement that "increased toxic substances in the blood as a consequence of liver damage could also have contributed to the neurological signs" should be ruled out in this particular case. The neurological signs that forced the euthanasia of this animal could be due to the severe lymphocytic meningitis observed (Figure 2 i), a clear indication of an intense inflammatory reaction, probably caused by the passage of the virus through the CNS. In order to avoid misunderstandings, the phrase about the possible role of toxic substances in the development of neurological signs has been deleted and the paragraph has been reworded as follows (new sentences added in red):

However, this animal showed no presence of viral antigen in the brain, neither in circulating cells nor in cells constituting the brain parenchyma, contrary to other studies in which neurological damage late after infection has been correlated with the presence of virus in the brain [15, 42, 44, 46]. Even though virus was not detected in samples from the brain and other organs, severe lymphocytic meningitis was observed (Figure 2 I), probably induced by the viral infection, that may have contributed to the neurological signs that forced the eutanasia of this animal. ~~It is also plausible that, as a consequenceof the liver failure caused by viral infection at the early stages, the increase of toxicsubstances in the Eveblood also contributed to the neurological signs.~~ In the absence of virus detection, organ lesions, or blood chemistry data, it is difficult to explain the signs that led to the sacrifice of this animal. The only objective sign of infection found in mice inoculated with 40Fp8 was the presence of virus in ~~Indeed,~~ liver samples from 40Fp8#2 mouse taken on day 5 in the second experiment ~~show the presence of virus in this organ~~, with a pattern of results …

Editorial and data presentation modifications

Reviewer #1: minor revisions, the paper could benefit from more robust statistical analyses and explanation of the public health relevance of these findings

Already discussed

Reviewer #2: The figures with microscopy images can have scale bar.

Scale bars have been added in Figures 2 and 4 (and included also in the new supplementary figure 2). In their corresponding legends the sentence

IHC, magnification: 10x and 40x

has been substituted by:

(Figure 2) *"IHC, scale bars: 50 micrometers (f, g, i); 200 micrometers (b, c, d, e, h)"*; (Figure 4) *"IHC, scale bars: 50 micrometers (c); 100 micrometers (insets: b,f); 200 micrometers (b,d,e,f,g)"*.

Reviewer #3: The Discussion in its present form needs to be reduced considerably. Lines 364 to 402 give background information that could be discarded without compromising the manuscript.

In accordance with the reviewer's suggestion, some background questions have been removed. However, we consider important to retain some information regarding the pathogenesis of RVFV strains in the mouse model, as this will facilitate a better understanding and highlight our findings to those less familiar with RVFV. Consequently, some paragraphs have been deleted and the discussion begins as follows:

The pathogenesis of virulent RVFV strains in the mouse model is characterised by rapid death of most animals 3-6 days after infection with severe hepatitis. Those that survive this acute onset of liver disease, despite viral clearance from visceral organs, succumb to late-onset encephalitis around day 9-10, with viral neuroinvasion and neurological signs [44-46]. Although this is not the usual pattern of RVFV infection in livestock, which is mainly characterised by reproductive failure and hepatic injury [1, 47], this dual hepatic-neurological disease is a typical outcome of severe RVFV infection in humans [2], making studies in mice particularly relevant.

The route of inoculation can lead to different patterns of virus dissemination with differences in the main organs targeted and the kinetics of viremia [52] . The intranasal (IN) route, that mimics one of the common routes of human infection when exposed to contaminated material [46] (reviewed in [51]), constitutes an extremely severe form of infection in rodents, even after inoculation with attenuated virus. It has been suggested that IN infection favours the entry of the virus into the central nervous system and thus the development of the neuropathologies described in RVF [15, 16, 53].

In this work we combined two extremely sensitive procedures using of A129 IFNARKO mice, that provide an extremely sensitive system for assessing the degree of viral attenuation. [32, 33] (for a review, see [50]), and the IN route of administration .....

Consequently, references numbered as 39, 40, 41, 43, 48, 49, 51, 54 and 55 in the original manuscript have been deleted and the section changed accordingly.

Minor edits:

Lines 58-60, revise so that the sentence makes sense.

The original sentence:

"Host susceptibility differs depending on the species and age, being sheep and in particular young animals the most susceptible, with deaths up to 90-100%"

has been reformulated :

"Host susceptibility varies with species and age. Sheep, and especially young animals with deaths up to 90-100%, are most susceptible"

Line 259, reference to Fig 1c seems inappropriate as this data is not HP or IHC.

Deleted

Line 260, state precisely how many mice.

The original sentence:

"*except for a few mice in the high dose group"*

has been changed to:

"except for 4 mice in the high dose group (M_H_2, M_H_3, F_H_1 and F_H_3, supplementary table 1 A)"

Line 410, spelling – demonstrated

corrected

Line 425, revise to "..with both false negatives and positive.."

The sentence has been changed as suggested

Line 426, spelling – extraction

corrected

Line 629, spelling – occasionally

corrected

Figure 4 legend, state the colour scheme used.

The next sentence has been included: *The borders of the bars have been coloured according to the virus inoculated: green: rG1; black: rMP-12; blue: 40Fp8.*

Summary and general comments

Reviewer #1: (No Response)

Reviewer #2: The present work by Borrego et.al. aimed to extensively test the attenuation of a live attenuated vaccine candidate 40Fp8 against Rift Valley fever (RVF) arbovirus, utilizing an extremely immunodeficient, interferon alpha receptor knockout (IFNARKO) mouse model with an aggressive intranasal inoculation route. The authors performed well thought out experiments by well-established methods to carefully assess the attenuation of 40Fp8 and compared this to previously well characterized and licensed live attenuated virus (LAV) strains having deletion in non-structural segments (NSs-) and another possessing twelve mutations in various genomic segments MP-12. The survival studies revealed, 40Fp8 infected mice at both high (10^3 pfu) and low (50 pfu) doses survived for extended times (MST: day 15) with mild to negligible clinical signs, minimal weight loss (high dose group), undetectable virus by qRT-PCR (Cq: 32-37) and replicating virus by IHC and serial passaging (up to three passages) of tissue harvests from major infection susceptible organs such as liver, brain, spleen, and kidneys providing strong basis for extreme attenuation and outweighing the vaccine effectiveness. In comparison, the other two LAV strains NS- and MP-12 infected mice at both the high (10^3 pfu) and low (50 pfu) doses succumbed to death relatively quickly (MST: day 4/5) characterized by detectable viral replication by qRT-PCR (Cq: 20-25), detectable virus by IHC in lesions and replicating virus by passaging (single passage) from major organ tissue harvests. Taken together these results indicate significant attenuation of 40Fp8, albeit with single incidences of mutifocal meningitis (day 9, experiment 1) without detectable viral antigen and in another experiment the authors recovered infectious virus from liver (day 5, experiment 2).

The claims by the authors are justified by their associated results with well thought out experiments with appropriate controls. The manuscript is well written and cited relevant literature.
The authors should address the following major concerns:

All the questions posed by the reviewer are of great interest, but unfortunately, the data and samples collected in the experiments described in this manuscript do not allow us to answer. Some of the points are already the basis for our current or planned research, while the others have provided us with new ideas to afford for future applications.

1. The manuscript lacks a vital data relating to the lungs of the infected mice given the intranasal route of inoculation. The authors must provide the IHC, qRT-PCR and immunofluorescence labelling of the mice lung tissue from each infection group.

   Although inoculation via the IN route may render the lungs an interesting tissue to analyse, it is well known that the liver is the main target of RVFV regardless of the inoculation route. To our knowledge, no development of respiratory disease or any

effect on lung infection pattern has been described associated with IN inoculation or aerosol exposure to RVFV. The virus is indeed detected in the lungs, but this does not appear to be the major target for infection (Reed et al., 2013; Bales et al., 2012; Brown et al., 1981). Conversely, it is well documented that the IN route may lead to changes in brain dissemination and higher hepatic and neurological damage. Consequently, we focused our analysis on the liver and brain, as most works do, and did not collect lung tissue. This will be taken into account in future experiments.

2. Given the concerns the authors proposed relating to escape of other LAVs given the endemic nature of the virus. The authors are encouraged to provide basis for non-susceptibility of 40Fp8 for escape given the rare yet probable infection of naturally circulating virus in endemic animal cohorts.

A common concern with genome-segmented viruses is that vaccination with LAVs in areas where the virus is circulating may lead to genomic reassortment and the emergence of new viruses that are no longer attenuated. However, the low (undetectable) levels of viremia induced by 40Fp8 vaccination allow us to suggest that this would be a low probability event. In any case, we agree with the reviewer that this is an important issue to be addressed. In fact, part of our current research is focused on investigating the attenuation conferred by each one of the 3 genomic segments of 40Fp8. Our results suggest that each of the segments, even separately, results in an attenuated virus. However these results are too preliminary to be reported or mentioned in the manuscript.

3. Acknowledging the challenges, it will be valuable addition to provide virus challenge studies with the replicative virus to show protective efficacy of the 40Fp8 challenged mice? The authors are encouraged to address why was it not included or is being tested? This data is going to be very helpful in providing even higher confidence associated with the present study results.

We agree with the interest in testing the protection conferred by the immunity induced by 40Fp8. Unfortunately, due to the restricted schedule of availability of BSL-3 boxes for RVFV animal experiments, the design of the experiments described here did not include challenge. Ethical issues compel us to reduce the number of challenge experiments classified as severe, and protection must be predicted on the basis of some immune parameters, such as the neutralising antibody levels, which are accepted as the clearest correlate of protection. As we mentioned in the original manuscript (lines 434-436), based on the results of our previous studies, it is likely that most animals in the 1000 pfu inoculated group would be protected. Anyway, experiments of 40Fp8 vaccination followed by virulent challenge are being carried out in wild-type, immunocompetent animals (both mice and adult sheep). We believe that this may provide more valuable insights, as the extreme attenuation of 40Fp8 may be a handicap for the induction of efficient immune responses. Results will be published soon.

4. Given the incidence of neuropathology's associated with 40Fp8. The authors are encouraged to provide information on the severity of higher 40Fp8 viral doses (>103 pfu) tested?

Again, in vivo RVFV experiments are constrained by the availability of BSL-3 boxes and ethical considerations as outlined in the manuscript (lines 460-466). The discussion has been slightly modified by adding a short sentence as follows:

"which would require a more accurate study with daily sampling of a significant number of animals inoculated with higher doses of virus" .

5. The authors are encouraged to address if the experiments with Balb/c mice passively blocking InfA (α-InfA) and infected with 40Fp8 were tested and if tested what were the results?

This would indeed be an interesting assay. The transient immunosuppression of the innate immune response should presumably render BALB/C mice susceptible to the infection with attenuated strains. We have not performed such experiments and we are not aware of this approach being done with other vaccine strains MP12 or NSs deleted viruses. We appreciate the suggestion and will consider it for future research.

6. Given that the virus was generated by serial passaging in the presence of favipiravir. The authors are encouraged to provide their insights into 40Fp8 vaccination in the context of pre/post favipiravir treatment of mice?

This is also an interesting question to address but it is not the focus of our current research so we can only speculate on the matter. Despite 40Fp8 being generated by serial passaging in the presence of favipiravir, its resistance to this antiviral was only partial, as higher concentrations of the drug abolished viral infectivity in cell culture (Borrego 2019 AAC). Consequently, 40Fp8 vaccination of mice under pre/post favipiravir treatment should consider the doses of drug administered and its pharmacokinetics. It is reasonable to speculate that the drug could exert some antiviral effect on 40Fp8, thus affecting its vaccine potential.

7. Further the authors are encouraged to comment on infection efficiency of 40Fp8 in various cell lines (eg: Vero)? It is clear from the authors previous work that 40Fp8 replication fitness is severed due to three mutations in the L gene. Further, it seems helpful if the infection efficiency of 40Fp8 were similar to its parental RVFV strain as the modified 40Fp8 expressing GFP could be potentially be used as substitute for virus neutralizations in BSL2 setting accelerating the testing / development of vaccine candidates for RVFV. The authors can provide insights into the same.

In our paper on the initial characterisation of 40Fp8 (Borrego & Brun 2021), we analysed its kinetics and total virus yield in 3 different cell lines: Vero, HEK293 and the C6/36 cell line derived from Ae.albopictus mosquito larvae. No significant differences were found in the 2 mammalian cell lines, whereas both virus growth and final yield were significantly reduced in C6/36 mosquito cells. Our current work is focused on the rescue of a recombinant r40Fp8 (and modified versions expressing reporter genes as proposed), to investigate the role of the mutations carried by 40Fp8 in these differences. The generation of further attenuated viruses is certainly an exciting goal with promising applications.

Following are a few minor comments:

1. Line 286: “data not shown”. The data can be included in the supplementary for clarity and transparency.

A supplementary figure with these images and the corresponding legend has been included (supplementary figure 2)

2. Line 351: immunolabelled.

corrected

Reviewer #3: The article reports the infection of INFAR KO mice with RVFV strain 40Fp8 to demonstrate the highly attenuated nature of this strain compared to other attenuated strains. Despite intranasal inoculation in a mouse strain lacking a functional interferon response, this strain was unable to cause disease although at high doses could induce an immune response. As the authors allude in the closing remarks of the manuscript, further studies are being attempted to evaluate strain 40Fp8 in ruminants.
Overall the data supports the authors conclusions although the Discussion should be reduced and a more convincing explanation for one death within the 40Fp8 high dose group is needed.

We thank the reviewer for the positive comments. With respect to the explanation for the unexpected death of a single animal in the high dose group, we remit to the comments on this same matter above.
